# Let Us Give Voice to Local Farmers: Preferences for Farm-Based Strategies to Enhance Human–Elephant Coexistence in Africa

**DOI:** 10.3390/ani12141867

**Published:** 2022-07-21

**Authors:** María Montero Botey, Mario Soliño, Ramón Perea, María Martínez-Jauregui

**Affiliations:** 1 Departamento de Sistemas y Recursos Naturales, Universidad Politécnica de Madrid, Avda. Moreras s/n E, 28040 Madrid, Spain; ramon.perea@upm.es; 2 Institute of Marine Research—CSIC, C/ Eduardo Cabello 6, 36208 Vigo, Spain; msolino@iim.csic.es; 3 Complutense Institute for International Studies (ICEI), Finca Mas Ferré, Edif. A. Campus de Somosaguas, 28223 Pozuelo de Alarcón, Spain; 4 Forest Research Centre (INIA-CSIC), Ctra. de La Coruña km. 7.5, 28040 Madrid, Spain; martinez.maria@inia.csic.es; 5 Sustainable Forest Management Research Institute, University of Valladolid and INIA, Avda. de Madrid 57, 34004 Palencia, Spain

**Keywords:** mitigation measures, choice experiment, human–wildlife conflict, *Loxodonta africana*, willingness to pay, beehives, chili-oil fences

## Abstract

**Simple Summary:**

Local communities living on the edge of protected areas often experience negative impacts on their livelihoods due to wildlife. These situations threaten support for long-term conservation of wildlife and wild habitats so a key for conservation sustainability should be based on implementing socially accepted and economically sustainable mitigation practices. For successful design and implementation of mitigation strategies, it is vital to engage local communities and understand their preferences and previous experiences. In this study, we present a choice experiment as a tool to analyze local farmer preferences for the most common farm-based solutions to reduce African elephant crop damage. Results show that there are significant differences among responses triggered by farmers’ previous experience with elephants and socioeconomic situation, with a marked spatial distribution among respondents. This methodology, based on a choice modeling approach considering the differential availability of resources and previous experience with elephants or other wildlife, is highly applicable, with small changes in other areas where wildlife competes with local communities for resources. This approach also represents a suitable instrument for identifying stakeholders’ preferences in each specific context.

**Abstract:**

Local communities surrounding wildlife corridors and natural reserves often face challenges related to human–wildlife coexistence. To mitigate the challenges and ensure the long-term conservation of wildlife, it is important to engage local communities in the design of conservation strategies. By conducting 480 face-to-face interviews in 30 villages along and adjacent to the Selous-Niassa Wildlife Corridor (Tanzania), we quantified farmers’ preferences for farm-based measures to mitigate African elephant damage using choice experiments. Results show that farmers considered no action the least preferred option, revealing that they are open to trying different measures. The most preferred management strategy matched with the preferences of wildlife rangers in the area, suggesting low concern about the potential conflicts between stakeholders. However, a latent class model suggests that there are significant differences among responses triggered by farmers’ previous experience with elephants, the intensity of the elephant damage, and the socioeconomic situation of the farmer. Results show a marked spatial distribution among respondents, highlighting the benefits of zone management as conflicts were found to be highly context dependent. Understanding the human dimension of conservation is essential for the successful planification and implementation of conservation strategies. Therefore, the development and broad utilization of methodologies to gather specific context information should be encouraged.

## 1. Introduction

Coexistence between people and wildlife has been long recognized as a global conservation challenge [1,2]. In some cases, coexistence with large-sized wildlife implies impacts on the safety or livelihood of local people. As a result, socio-economic conflicts may arise, confronting local communities negatively affected by the presence of certain species and those who want to promote or protect those species [3]. Although people and wildlife have co-existed for millennia, wildlife-related conflicts have become more intense and frequent in recent years due to habitat loss and degradation, mainly caused by the expansion and intensification of human activities [4,5]. Africa is a paradigmatic example of increased conflicts related to wildlife due to the charismatic and threatened species involved, the recent growth of its human population [6], and the strong economic vulnerability of rural areas [7].

Compensation policies, where the government or conservationists pay for the damages occurred due to wildlife, may seem a good strategy to address human–wildlife conflicts [8,9,10]. However, the conservation of wildlife in Africa is generally encouraged by governments or organizations that are heavily dependent on outside sources of funding. Compensation policies are not advised in areas with limited funds or deficient administrative controls due to possible fraudulent claims and damage of the motivation of local communities to protect their properties from wildlife damage [11,12].

Previous research has shown that management tools to promote human–wildlife coexistence should consider not only the research on technical solutions but the development of shared solutions, where conflicting parties are engaged and cooperate [13]. This highlights the importance of co-management in addressing human conflicts with wildlife in Africa, where engagement of local communities is necessary for the implementation of successful and economically sustainable mitigation strategies in the long term [14,15].

Empowering farmers to implement simple farm-based but cost-effective measures [16] could be a particularly successful alternative to mitigate conflicts in African wildlife corridors. In these areas, conservation programs are necessary for the maintenance of wildlife meta-population processes [17] and connectivity [18]; however, wildlife shares land and resources with rural communities, triggering important social costs [19]. Although government and private financial support is frequently scarce, it is already known that affected farmers are more willing to accept changes they have chosen themselves [20,21]. Similarly, the context and experiences farmers have accumulated during their lives have been identified as key factors to engaging farmers in mitigation practices in Asia [22,23]. Therefore, the incorporation of farmers’ preferences for different farm-based measures and their relationship with farmers’ previous experiences is urgently needed for the successful and context-dependent design of wildlife conservation programs.

In this study, we used tools from environmental economics to address preferences among farmers in the Selous-Niassa Wildlife Corridor (Tanzania) related to: (i) the specific farm-based measures they consider effective in preventing African elephant (*Loxodonta africana* Blumenbach 1797) damage and their willingness to apply them, (ii) the importance of receiving technical advice (conducted by NGOs or the Government) in the implementation of the measures, and (iii) the desirable level of cooperation in their community for this implementation (which was proven to be a key factor in the success or failure of human–elephant conflict mitigation programs in other areas, e.g., [16]). To avoid false expectations being raised in the local communities, all proposed strategies are supported by science, relatively inexpensive, and applicable by the farmers on their own. In addition, wildlife rangers were informed about these strategies and their preferences were previously analyzed [24], which will allow us to shed some light on the potential conflicts between rangers and farmers when choosing, planning, and implementing the proposed mitigation measures. Conflicts between rangers and farmers regarding the implementation of mitigation measures influence the success of the measures as wildlife rangers hold a key role in the community awareness and protection of people’s livelihoods from wildlife [25,26]. These conflicts can also undermine trust and cooperation between the parties, influencing the implementation and success of other conservation activities [27], as rangers are, in many cases, the most visible actors in conservation to local communities [28].

Finally, and for a better understanding of the local communities’ preferences, including an analysis of the heterogeneous preferences among respondents [29] and its possible causes, we explored whether there are differences among responses triggered by farmers’ personal previous experience with elephants, either on their own farms or through family, friends’, or neighbors’ experiences (contagious effect of risk perception, [30]). Moreover, we also explored whether the actual socioeconomic situation of the respondents (measured by the self-reported food insecurity level) influences their preferences for the proposed measures. This exploration is important to identify factors that can influence preferences in other contexts.

## 2. Materials and Methods

### 2.1. Farming and Elephant Conservation in the Selous-Niassa Wildlife Corridor

**Figure 1:**
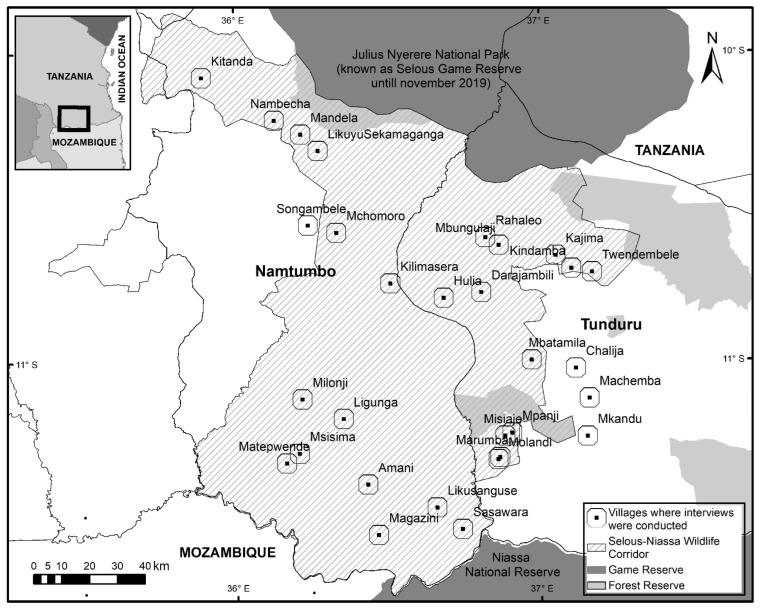
Selous-Niassa Wildlife Corridor map and location of villages where interviews were conducted.

The Selous-Niassa Wildlife Corridor (Figure 1) is part of the world’s largest Miombo woodland ecosystems (Selous-Niassa ecosystem) and links Julius Nyerere National Park (established in November 2019 but previously known as Selous Game Reserve) in Tanzania with Niassa National Reserve in Mozambique. The corridor lies within the Tunduru and Namtumbo districts in Ruvuma Region (southern Tanzania), covers traditional elephant movement routes [31], and harbors a population of 602 ± 258 elephants [32]. It is located entirely on the land owned by 30 villages. Local people mostly base their economy on subsistence farming, although this is more pronounced in the north part of the corridor. The staple crops grown are maize, rice, and cassava while common cash crops are tobacco, sunflower, cashew nut, sesame, etc. [33,34].

For local communities all around Africa, cohabitation with elephants commonly implies crop losses, damages to infrastructures and water supplies, and, in few cases, injuries or human deaths due to elephants charging at humans [35,36,37,38,39,40]. These situations disrupt the psychological and physical wellbeing of local communities [41,42,43] and involve many challenges for elephant conservation [44,45], fueling both legal and illegal retaliation killings of elephants [39,46,47] and threatening the maintenance of protected areas in the long term due to increased resistance to conservation [39,48]. In addition, damages have increased in the last century due to the rapid growth of the human population and the colonization of natural areas for its conversion into agriculture land [49,50], spreading all over the African elephant range [51,52].

The current decrease in the elephant populations in the Selous-Niassa ecosystem [53] and the rise in impacts on humans lives due to frequent human–elephant interaction [54] make the area a unique place to address large-scale human–elephant coexistence challenges and establish sustainable local initiatives for the mitigation of conflicts related to farming and wildlife conservation. Additionally, Tanzania is an example where the government and local communities are willing to engage in mitigating these types of challenges. This is proved by the “National Human-Wildlife Conflict Management Strategy 2020–2024” [54] and the fact that rangers commonly work on chasing away elephants from farms and are also involved in citizen science [24]. In addition, some farmers are already applying some farm-based mitigation measures, such as chili fences, encouraged and supported in the corridor by PAMS (Protected Areas Management Solution) Foundation and WWF (World Wild Fund for Nature). However, in the Selous-Niassa Wildlife corridor, the most common elephant mitigation measures applied by farmers are guarding the crops at night and making noises to chase them away (drumming, clapping, shouting, etc.), which are traditional methods that they have broad knowledge of and do not represent an added cost to their already vulnerable and limited familiar economy.

### 2.2. Data Collection

Data was collected by conducting 480 face-to-face interviews in 30 villages along and adjacent to the Selous-Niassa Wildlife Corridor (Figure 1). The sampling unit was the household. Households were chosen randomly, and interviews were restricted to one respondent (above 18 years old) per household. In each village, 16 locals were interviewed, 8 men and 8 women, in equal proportions between people interviewed in the village center and in further farms inside the village land. All interviews were conducted between June and September 2019 in Swahili by five previously trained Tanzanians from the area. The survey was pre-tested in April 2019 on 25 farmers from 3 villages with different intensities of elephant damage to ensure clarity before use and improve the design of the final study. The questionnaire (Supplementary File A) was designed to gather four categories of information: (1) personal data (gender, occupations, food shortage in their household, etc.), (2) previous experience with elephants and elephant crop damage, (3) perception of the effectivity of farm-based elephant mitigation measures using Likert scales (from 1 to 4, where 1 represented strongly disagree, 2 disagree, 3 agree, and 4 strongly agree; don’t know was always available for the respondent), and (4) preferences for mitigation tools and their implementation using a discrete choice experiment [55].

### 2.3. Choice Modeling

To analyze the local communities’ preferences regarding farm-based management programs, we designed a discrete choice experiment (DCE) composed of four attributes. The DCE is a stated preferences method that involves presenting respondents with various choice cards comprising two or more alternatives (actions, programs, scenarios, etc.) that are described by a set of attributes and different levels. This method is commonly used to obtain comparable measures of preferences across factors and attributes [56,57].

**Figure 2:**
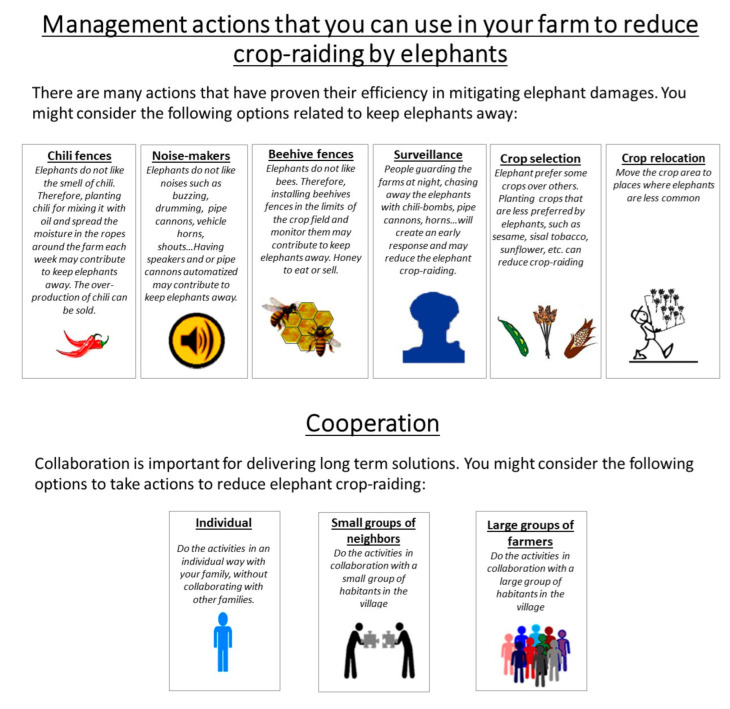
Examples of explanatory cards showed to the interviewees to define the specific farm-based measures that farmers can apply to reduce elephant damage and the level of cooperation in the implementation of those measures.

The attributes were equal to those employed in the rangers’ preference exploration in the same study area [24]. They are: (1) specific farm-based measures that farmers can apply to reduce elephant damage to humans and human means, which include six different scientifically proven effective strategies: (a) chili-oil fences [58,59,60]; (b) noisemakers [61,62]; (c) beehive fences [63]; (d) surveillance [61,64]; (e) crop selection [34,65,66,67]; and (f) crop relocation [68]; (2) the level of cooperation in the implementation of different tools, which has been defined as an important key for the success of mitigation measures [16], defined in a qualitative manner: (a) individual, (b) small groups of neighbors (2–3 households, as represented in Figure 2), and (c) large groups (>10 households, as illustrated in Figure 2) and community levels) [69,70]; (3) the involvement of technical support given by NGOs or the government in the process [71] considering (a) yes, it is present, and (b) no, it is not, which provides important information about how much farmers trust those institutions; and (4) a monetary attribute to estimate the willingness to pay per household and commonly used to quantify preferences. In this case, we also considered the monetary cost that farmers should assume when implementing the elephant crop damage mitigation program, which was not considered in the rangers’ study performed by Montero-Botey et al. [24]. The monetary attribute had four levels from 10,000 TZS (~5$) to 40,000 TZS (~20$) and represented the monetary cost per year for a farmer to apply the measure selected in one acre. The levels of cost were established after a discussion in a focus group with members of the community to determine the range of cost that farmers would be willing to invest and could afford as the majority are subsistence farmers. It was also tested in the pilot questionnaire. A more extensive description of the first two attributes is available in Figure 2.

**Figure 3:**
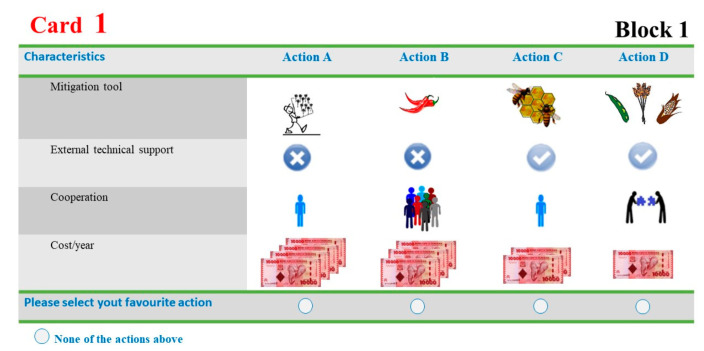
Example of a choice card used in the DCE.

Based on the results obtained by the pilot study of 25 farmers in the study area, a D-efficiency criterion to generate efficient designs was considered to identify the lower D-error that minimizes the variances and covariances of the parameter estimates [72]. We used the Ngene^®^ 1.2. software [73] for our experimental design and 48 choice cards were generated. In order to make a feasible choice task, and not overwhelm the respondents with too many choices, a blocking strategy was considered, and twelve choice cards were shown to each individual. Each choice card comprised four alternative programs and an opt-out option that represented a no-intervention alternative to avoid forcing activity choices [74] (Figure 3).

The final data of farmers’ choice was analyzed in two steps. First, for comparison with the wildlife rangers’ preferences reported in Montero-Botey et al. [24], we estimated a random parameters logit model using the Nlogit^®^ version 6 software. We assumed that all the attributes are random parameters that are normally distributed and the willingness to pay (WTP) for each attribute level was estimated (see the formulation in Supplementary File B).

Secondly, we estimated a latent class model (LCM) with random parameters [75,76] using the Latent GOLD^®^ version 5.1 software [77] (see the formulation in Supplementary File B). This modeling approach is useful for the in-depth analysis of heterogeneous preferences among respondents [29], possibly associated with previous experience with elephants [78] and the possible social contagion of risk perception [79]. For this purpose, we created an artificial variable classifying the farmers directly affected by elephant crop damage; farmers not directly affected by elephant crop damage but whose family, friends, or neighbors have been affected; and farmers not affected without relatives or neighbors affected by crop damage. Based on the results from the latent class model, we carried out a post-hoc descriptive analysis to show the spatial distribution of the classes as zoning management could improve the achievement of conservation goals [80]. We also explored the relationship of those classes with food shortage and elephant presence as indicators of vulnerability [30].

## 3. Results

A total of 241 men and 239 women were interviewed: 95% of them focused on agriculture as their main occupation and 78% were originally from the village where they were interviewed. Elephants were considered the most conflictive wildlife species in the area by 76% of the respondents (see more information in Table S1). Regarding their personal experience with elephants, 75% had seen an elephant, 4 people reported to have been directly charged by elephants, 9% that family members or friends were charged, and 13% that the closest person charged they know about was someone from their village.

**Figure 4:**
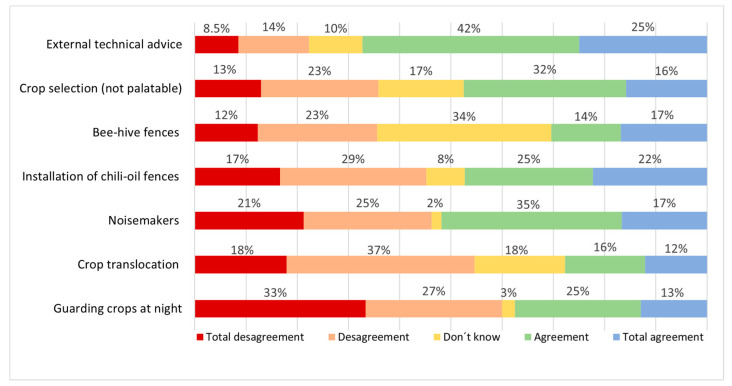
Farmers’ perception about the effectivity of farm-based mitigation measures to reduce crop damage by elephants.

Regarding elephant crop damage, 55% of them reported that they had been directly affected (average of 4 times in their lifetime), 12% that not them but their family or friends had been affected, and 8.5% that the closest person affected they knew about was someone from the village they live in. Concerning the perceived effectivity of measures to reduce crop damage (Figure 4), noisemakers were considered effective by 52% of respondents (2.48 ± 0.05 in the same Likert scale, from 1 to 4), crop selection by 48% (2.6 ± 0.04), chili-oil fences by 47% (2.57 ± 0.05), guarding crops at night by 38% (2.18 ± 0.05), bee-hive fences by 31% (2.53 ± 0.05), and crop translocation by 28% (2.27 ± 0.04). Technical advice was considered effective by 67% (2.93 ± 0.04). Importantly, 34% did not know about the bees as a mitigation measure and 17% and 18% were not sure about the effectivity of crop selection and crop translocation, respectively.

Choice experiment results showed that farmers in the Selous-Niassa Wildlife Corridor generally agreed with a farm-based management program to mitigate elephant crop damage. However, 2.5% did not choose any option due to budgetary restrictions (true zeros) and 4.6% (protest responses) refused to choose options in the choice experiment due to other reasons such as, for example, that the mitigation measures should be implemented and paid for by the government and/or the lack of elephants in their area. For the rest of the respondents that made any choice (93%), the option “no action” was chosen in 11.5% of the observations. For the analysis of preferences, we excluded the protest responses (4.6%), and the final sample was composed of 27,420 observations of 457 individuals. Results showed that the alternative specific constant (ASC) was statistically significant (Table 1 and Table S2).

The results of the random parameters logit model (Table 1) show that regarding the mitigation tools, farmers’ most preferred tool was the use of chili-oil fences, followed by bee-hive fences, having technical support, promoting cooperation in large groups (community levels), and crop selection. Using noisemakers and surveillance and cooperation in small- and medium-sized groups were not significant, and translocating crops was rejected as it reduces overall farmers’ well-being.

**Figure 5:**
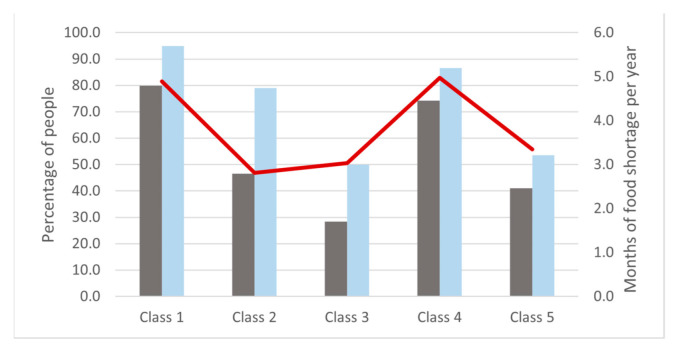
Description of the classes regarding the percentage of respondents that had seen an elephant (blue bar) and the percentage of respondents that had suffered a food shortage in their households (grey bar). The line shows the average duration of the food shortage period (in months). Class 1: Affected and cooperative; Class 2: Not affected and cooperation in small groups; Class 3: Not affected and communal; Class 4: Affected and individualist; Class 5: Not affected whose family, friends, or neighbors have been affected and individualist.

**Figure 6:**
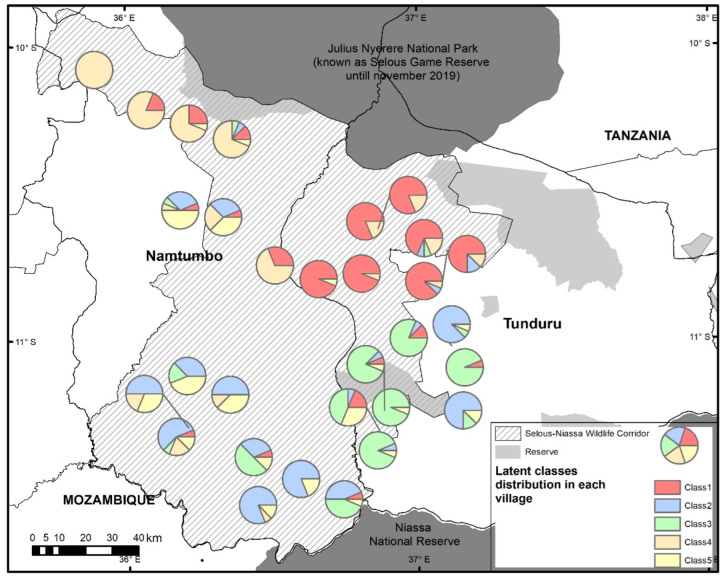
Latent classes’ distribution by village in the Selous-Niassa Wildlife Corridor. Class 1: Affected and cooperative; Class 2: Not affected and cooperation in small groups; Class 3: Not affected and communal; Class 4: Affected and individualist; Class 5: Not affected, whose family, friends, or neighbors have been affected and individualist.

The latent class model identified five different classes of behavior among the respondents that explained the mitigation strategies’ choice heterogeneity (Table 2). Combining this information with their experience, food shortage (Figure 5), and the geographical context (Figure 6 shows the spatial distribution of every class in the villages of the Selous-Niassa Wildlife Corridor), we characterized and further explained the classes that resulted from the model. The main findings are: (i) 24.1% of the respondents (class 1) were directly affected by elephants, had suffered from a severe food shortage, and were willing to cooperate at the village level; (ii) 23.8% of the respondents (class 2) were not directly affected by elephants, had suffered a moderate food shortage, were not concerned about the economic cost, and were not very demanding on the characteristics of the program; (iii) 21.1% of the respondents (class 3) were not directly affected by elephants, suffered a lower food shortage, were willing to pay much more (almost 6-fold), but they were in favor of a program involving the whole community and technical support; (iv) 18.5% of the respondents (class 4) were directly affected by elephants, had suffered from a severe food shortage, valued technical support but they preferred an individual program, and had a strong negative reaction to crop translocation; (iv) and 12.5% of the respondents (class 5) were not directly affected and were characterized by low cooperation and strong willingness to pay (almost 4 times more than the directly affected classes; Table 2), with little or no apparent value for technical support.

## 4. Discussions

Our results show that most farmers see at least some viability in local-based initiatives to reduce elephant coexistence challenges at the farm level and are willing to invest in these measures. The fact that they considered no action the least preferred option (only selected 4.6% of the time) reveals that they are open to trying different measures. This reveals a promising future for elephant conservation as local communities represent the key level to applying elephant mitigation measures, particularly when financial support is limited [15,16,81]. The involvement of local farmers will also enhance the engagement and feeling of ownership, which is a critical aspect to ensuring its success and long-term sustainability [82,83]. We found that the willingness to be involved in mitigation programs could be at least partly explained by the high percentage of people directly affected (55%) by elephants, their food shortage period, and their geographical context.

### 4.1. General DCE Results: Farmers’ vs. Rangers’ Preferences

Using a discrete choice experiment, we found that the most suitable program according to farmers’ preferences, in general, should consider chili-oil fences, cooperation in large groups (community levels), and the availability of technical support. This result agrees with the ranger preferences analyzed in the same area [24]. Acceptance, willingness to apply, and agreement between stakeholders are important assets for the successful implementation of a project [14].

Chili-oil fences have been found to be a useful tool in keeping elephants away from farms in some African regions [59,60]; however, other studies have found them to be not that effective [84]. In the study area, few farms have been using this method in the last 10 years with inconclusive results as many of the farmers fail to maintain the fences at the needed frequency due to a lack of awareness and high costs [85]. The willingness to cooperate with other farmers at the community level suggests that they are aware of the advantages that cooperation can bring [69,70], although it could also be due to cultural reasons [86]. This could represent major progress as the lack of cooperation inside local communities has been identified as one of the main causes of failure for programs aiming to reduce human–elephant conflicts in other areas [16]. On the other hand, the high value that farmers gave to receiving technical support [71] will likely benefit both farmers and elephants.

Although chili-oil fences are considered a good strategy, it has greater chances of succeeding if it is combined or rotated with other elephant mitigation measures [81,87,88]. Therefore, understanding rangers’ and community preferences regarding other less-preferred measures is still important. The other two most-preferred measures by local communities were bee-hive fences and crop selection (i.e., growing less palatable crops such as sesame, sunflower, or chili; [24,89]). Previous research [63,69,89] has found these measures to be effective in reducing elephant damage in other regions of Africa. However, these measures have never been systematically applied along the corridor and, therefore, are less known by farmers. Although both measures were also highly valued by rangers, farmers preferred bee-hive fences over crop selection because shifts to less palatable crops may represent an important challenge to the communities [34], as they will become more dependent on external food sources [90,91].

Our results reveal that noisemakers (proved effective by previous research [61,62,64,92,93], although with some concerns about its long-term effectivity in the case of audio playbacks due to elephants getting used to them [87]), surveillance and cooperation in small groups were not significantly preferred, although they are commonly used in the area and were considered effective by most of the respondents in previous perception questions. Finally, we should point out that crop relocation usually decreases farmers’ wellbeing and it was, therefore, not preferred by farmers and less preferred by wildlife rangers than other options [24]. However, it is important to highlight that 4% of the respondents, all coming from villages with a high intensity of elephant crop damage, directly asked for help in convincing their fellow farmers to organize block farming initiatives to facilitate the implementation of mitigation measures. This also suggests that the differential preferences and attitudes of farmers could be motivated by the intensity of elephant crop damage. We highlight that future studies should consider the intensity of the elephant damage and the resources available in each area by including, for instance, the drought periods and potential food shortage (intensity and duration). This is important because water availability influences elephant movements [94] and the intensity and probability of crop damage [34,95]. In addition, droughts have a negative impact on farmers’ food security [96] and increase the competition between wildlife and people for water resources.

### 4.2. Great Heterogeneity among Farmers

As the latent class model showed, the DCE results present high heterogeneity in the preferences of the farmers linked to previous experience with elephant crop damage. The model presents a marked spatial distribution of the classes, which highlights the benefits of zoning management [80,97] and suggests that preferences might change due to other potential causes such as the intensity of the damage, previous experience with animals, and farmers’ socioeconomic situation [98,99]. This aligns with previous research stating that the management of conflicts related to wildlife should be context-specific [1,100]. Classes 1 (affected and cooperative) and 4 (affected and individualistic) cover the preferences of most of the farmers that have been directly affected by elephants, who are mostly located in the northern part of the corridor. The northern part of the corridor borders Julius Nyerere National Park and elephants’ incursions onto village land are common, and particularly frequent on the Tunduru side. Although it is surprising that they are willing to pay less than farmers that have not been affected by elephants on their farms (in contrast with previous research such as [22,23]), this can be easily explained by the high level of food shortage they reported and, therefore, the lack of resources. In addition, their previous experience with elephants probably makes them more realistic in their choices. However, it is very important to highlight that in both classes, Class 1 and 4, although they differ in the preferred level of cooperation, they highly value receiving technical support, appreciating the work of the government and NGOs.

Other areas (e.g., southern Tunduru, Class 3—not affected and communal) have recently reported a rise in elephant crop damage. This lack of previous experience together with a less extreme socioeconomic context may explain their remarkable concern, their value of technical support, and their willingness to pay and implement mitigation measures. They have recently been exposed to a threat, and it is likely that they are more fearful of elephants than other farmers who have previously experienced them as reported, for example, in other human–wildlife conflicts, such as wolves [101] or other animals [102,103]. This seems to indicate that the novelty or long-term duration of the exposure to elephant crop damage also affects farmers’ preferences for and involvement in mitigation measures.

Experience with a natural hazard influences risk perception [104,105] and the motivation to protect against future events [106]. Although farmers affected by elephants have been identified as more willing to apply elephant crop damage mitigation measures in Asia [22,23], our results show that having suffered a hazard (Class 1 and 4) does not always leads to a greater willingness to invest in the implementation of mitigation measures [107] in a food shortage context and might encourage some measures but not others [108].

Instruments for identifying stakeholders’ preferences considering the differential availability of resources and previous experience with wildlife, such as the methodology presented in this study (choice modeling), represent an important tool and could be broadly applied as a first step to design successful conservation and mitigation strategies in areas where wildlife competes for resources with local communities. They not only provide valuable insights for the creation of cohesive strategies but help in the identification of areas where common and new mitigation strategies, e.g., the smelly repellent [109], can be most successfully trialed or implemented within a landscape. They may also lead to further exploration of the reasons why the implementation of a perceived effective measure is less preferred in some places but not in others, and the possibility of increasing the willingness of implementation by planning side activities such as incentives, training, etc.

Studying local communities’ perceptions and preferences should be a must for implementing long-term programs related to wildlife, particularly in large extensions where no strong government and private financial support is available. When decisions respond to local stakeholders’ preferences, the perceived legitimacy of those conservation activities is typically increased [20,21,110]. Thus, understanding the human dimension of conservation is essential to ensuring effective communication, long-term implementation, and an integrative evaluation of conservation programs [111].

## 5. Conclusions

Economic valuation methods allow the determination of farmers’ preferences for farm-based strategies to reduce crop loss due to elephants. These preferences are context-specific as farmers’ preferences are influenced by previous experience of damage and socioeconomic factors, such as food shortage or drought length, which are also closely linked to their geographical context.

Gathering local communities’ perceptions and preferences and including communities in decision making is an important step in designing conservation strategies as it enhances the engagement, legitimacy, and feeling of ownership of these strategies in the community. We found that context dependence is a crucial, often overlooked component of coexistence management solutions, where appropriate measures vary in space and time according to local history and overlapping challenges. Therefore, the development and broad utilization of methodologies to gather context-specific information should be encouraged as an important tool in the design of mitigation measures, particularly in cases where elephants and communities share land. Successful implementation of these measures within the landscape will be key to ensuring elephant conservation and a peaceful coexistence with humans in the long-term future.

## Figures and Tables

**Table 1:** Results of the random parameter logit models (457 face-to-face wildlife rangers and 12 choices per individual; number of observations = 5484; Log likelihood function = −6410.09; restricted log likelihood = −8826.16; McFadden Pseudo R-squared = 0.2737; replications for simulated probs. = 500; used Halton sequences in simulations).

	Coefficient	Standard Error	Z	Prob. |z| > Z *	95% Confidence Interval
Random parameters					
ASC	−2.485 ***	0.2080	−11.94	<0.001	(−2.8927, −2.0772)
Crop selection	0.288 **	0.1226	2.35	0.019	(0.0478, 0.5286)
Crop translocation	−0.38 7 ***	0.1135	−3.41	<0.001	(−0.6092, −0.1645)
Noisemakers	−0.075	0. 1332	−0.57	0.571	(−0.3365, 0.1858)
Chili-oil fences	1.213 ***	0.1131	10.72	<0.001	(0.9908, 1.4343)
Bee-hive fences	0.708 ***	0.1257	5.63	<0.001	(0.4617, 0. 9545)
Technical support	0.658 ***	0.0748	8.79	<0.001	(0.5111, 0.8044)
Cooperation in small groups	−0.024	0.0603	−0.39	0. 694	(−0.1419, 0.0944)
Cooperation in big groups	0.437 ***	0.0604	7.24	<0.001	(0.3189, 0.5557)
BID Cost/year	−0.110 ***	0.0081	−13.60	<0.001	(−0.1254, −0.0938)
Standard Deviations of random parameters (normally distributed)					
ASC	3.091 ***	0. 1862	16.60	<0.001	(2.7263, 3.4562)
Crop selection	1.741 ***	0.1204	14.46	<0.001	(1.5047, 1.9766)
Crop translocation	1.243 ***	0.1115	11.16	<0.001	(1.0248, 1.4617)
Noisemakers	1.865 ***	0.1580	11.80	<0.001	(1.5557, 2.1751)
Chili-oil fences	1.8465 ***	0.1009	18.30	<0.001	(1.6487, 2.0443)
Bee-hive fences	2.070 ***	0.1337	15.48	<0.001	(1.8080, 2.3321)
Technical support	1.238 ***	0.0708	17.49	<0.001	(1.0992, 1.3766)
Cooperation in small groups	0.218	0.1621	1.34	0.180	(−0.1001, 0.5351)
Cooperation in big groups	0.5142 ***	0.0823	6.25	<0.001	(0.3530, 0.6754)
BID Cost/year	0.135 ***	0.0070	19.26	<0.001	(0.1217, 0.1492)

*** Significance at 1% level; ** Significance at 5% level; * Significance at 10% level.

**Table 2:** Results of the latent class model of respondents’ preferences for management tools.

	Class 1		Class 2		Class 3		Class 4		Class 5	
	Affected and Cooperative		Not Affected and Cooperation in Small Groups		Not Affected and Communal		Affected and Individualist		Not Affected Whose Family, Friends, or Neighbors have been Affected and Individualist	
Variable	β	θ	β	θ	β	θ	β	θ	Β	θ
Crop selection	−0.29	1.44 ***	1.27 ***	1.99 ***	1.34 ***	1.10 ***	−0.16	−0.47	3.37 ***	−0.29
Crop translocation	−0.63 **	2.08 ***	0.73 ***	0.66 ***	0.18	0.02	−3.85 ***	9.09 ***	−3.07	−0.63 *
Noisemakers	0.01	0.52 ***	1.42 ***	1.42 ***	−2.81 ***	−2.92 ***	−1.41	4.67 ***	1.33 ***	0.01 **
Chili-oil fences	1.08 ***	1.27 ***	1.44 ***	1.83 ***	2.40 ***	1.83 ***	−0.88	11.11 ***	4.24 ***	1.08 ***
Bee-hive fences	0.43 **	1.03 ***	1.97 ***	1.97 ***	1.20 ***	2.94 ***	2.50 ***	3.93 ***	1.10 ***	0.43
Technical support	0.58 ***	0.08	−0.09	−0.30 ***	2.97 ***	0.64 ***	1.02 ***	−1.66 ***	0.10	0.58
Coop. small groups	0.47 ***	0.08	0.58 ***	−0.09	−0.65 ***	−0.61 ***	−1.04 **	1.57 **	−0.29 *	0.47
Coop. big groups	1.32 ***	−0.25 *	0.22 **	−0.30 **	0.82 ***	0.02	−0.79	1.84 **	−0.34 *	1.32
Cost	−0.13 ***	−0.05 ***	0.00	−0.06 ***	−0.05 ***	−0.04 ***	−0.63 ***	0.23 ***	−0.05 ***	−0.13 ***
	Class1		Class2		Class3		Class4		Class5	Overall
Class size	0.24		0.24		0.21		0.19		0.12	0.24
R²	0.21		0.12		0.48		0.71		0.47	0.21
R²(0)	0.20		0.17		0.50		0.82		0.48	0.20

The β is mean coefficient and θ is SD of random parameters. Significance: ***, 1% level; **, 5% level; *, 10% level.

## Data Availability

Data available upon request to the authors.

## Supplementary Materials

The following supporting information can be downloaded at: https://www.mdpi.com/article/10.3390/ani12141867/s1, Supplementary File A: Questionnaire. Main questions related to previous experience with elephants and crop damage mitigation measures; Supplementary File B: Formulations; Table S1: Principal characteristics of respondents in the Selous-Niassa Wildlife Corridor; Table S2: Willingness to pay. Results of the random parameter logit model.
