# Supplementary material for: Let Us Give Voice to Local Farmers: Preferences for Farm-Based Strategies to Enhance Human–Elephant Coexistence in Africa
Source: Animals (Basel). 2022 Jul 21;12(14):1867. doi: 10.3390/ani12141867 (PMC9311559; doi:10.3390/ani12141867)
Supplement: Supplementary file 1 [file animals-12-01867-s001.zip › animals-1814890-Supplementary File A.pdf]

Questionnaire. Main questions related to previous experience with elephants and crop damage mitigation measures.

Personal information:

Village ________________________ □ Farms □ Village center

Gender: □ Female □ Male

How old are you? ____________________________

What is your main activity or principal source of income? _____________________

Does your home usually suffer from a period of food shortage?

□ Yes, In which months?:

January	February	March	April	May	June	July	August	September	October	November	December
□	□	□	□	□	□	□	□	□	□	□	□

□ Yes, In which months?:

□ No

What is your opinion on the following elephant management actions. Are they effective to reduce elephant crop-raiding?

	Strongly disagree	Disagree	Agree	Strongly agree	Don't know
Installation of Chili-oil-covered fences	□	□	□	□	□
Using noise-makers (voices, sirens, …)	□	□	□	□	□
Beehive fences	□	□	□	□	□
Crop selection	□	□	□	□	□
Crop relocation	□	□	□	□	□
Guarding crop fields at night	□	□	□	□	□
Relocation of people away	□	□	□	□	□
External technical advice	□	□	□	□	□

Personal experience with elephants:

Do you know someone who has ever been charged by an elephant?

□ No
□ I´ve heard about someone
□ Yes, someone from this village
□ Yes, a friend or a neighbor
□ Yes, a close family member
□ Yes, Me

Do you know someone who has been involved in an elephant crop-damage situation?

□ No
□ I´ve heard about someone
□ Yes, someone from this village
□ Yes, a friend or a neighbor
□ Yes, a close family member
□ Yes, Me

In the last elephant crop-damage situation, were they using any kind of deterrent method against elephants?

□ Yes. Please, specify: ______________ □ No □ I don´t know
