# Supplementary material for: Let Us Give Voice to Local Farmers: Preferences for Farm-Based Strategies to Enhance Human–Elephant Coexistence in Africa
Source: Animals (Basel). 2022 Jul 21;12(14):1867. doi: 10.3390/ani12141867 (PMC9311559; doi:10.3390/ani12141867)
Supplement: Supplementary file 1 [file animals-12-01867-s001.zip › animals-1814890-Supplementary File B.pdf]

In the Discrete Choice Experiment, the individual farmers i indirect utility function (Vi) is represented by an additive linear expression:

$$V_{ij} = \alpha_j + S_{ij}\overline{\beta} + S_{ij}\theta_i + \varepsilon_{ij} \tag{S1}$$

where S_{ij} is the vector of the DCE attributes described in material and methods section, θ_i represents the deviations on individual preferences with respect to the mean values, and ε_{ij} is an i.i.d. type I extreme value random component. Coefficients β vary in the population with density $f(\beta|\Omega)$, with Ω denoting the parameters of density, i.e. $\beta_i = \overline{\beta} + \theta_i$. The probability of farmers i's observed sequence of choices $[y_1, y_2,...,y_T]$, assuming unitary scale parameters, is calculated by the integral:

$$P_i[y_1, y_2, ..., y_T] = \int ... \int \prod_{t=1}^{T} \left[\frac{e^{(\alpha_j + S_{ij}\beta_i)}}{\sum_{k=1}^{J} e^{(\alpha_k + S_{ik}\beta_i)}} \right] f(\beta | \Omega) d\beta \tag{S2}$$

where j is the alternative chosen in choice occasion t.

For getting a more detailed analysis of heterogeneity among farmers, a Latent Class Model (LCM) is generated. Individual's preferences are represented by $\beta_i = \overline{\beta} + \theta_i$, but in this case the distribution $f(\beta)$ is discrete, with $\overline{\beta}$ taking a finite number of classes (k=1,…,K), so β_{ik} follows a distribution with density $f(\beta)$ for each class k. Therefore, the probability of individual observed sequence of choices [y1,…, yT] is simulated, as it follows:

$$P_i[y_1, ..., y_T] = \sum_{k=1}^{K} F_{ik} \frac{1}{R} \sum_{r=1}^{R} \left[\prod_{t=1}^{T} \frac{e(.)}{\sum_{h=1}^{J} e(.)} \right] \tag{S3}$$

From the observed choices, individuals' preferences are transformed into willingness to pay (WTP) for the attributes. The WTP for each level *k* of the attribute *j* (excluding the reference level, which is assuming to be zero) is estimated using the formula:

$$\mathrm{WTP}_k^j = -\frac{\beta_k^j}{\beta_{cost}} \tag{S4}$$
