# Supplementary material for: Let Us Give Voice to Local Farmers: Preferences for Farm-Based Strategies to Enhance Human–Elephant Coexistence in Africa
Source: Animals (Basel). 2022 Jul 21;12(14):1867. doi: 10.3390/ani12141867 (PMC9311559; doi:10.3390/ani12141867)
Supplement: Supplementary file 1 [file animals-12-01867-s001.zip › animals-1814890-Table S1.pdf]

Table S1. Principal characteristics of respondents in the Selous-Niassa Wildlife Corridor.

Class	N=480
Gender:	
Female	239
Male	241
Age (years):	
18-30	77
30-40	158
40-50	129
50-60	71
>60	45
Main occupation	
Agriculture	455
Livestock keeping	14
Teacher	9
Not mentioned	2
Originally from	
Same village where it was interviewed	376
Another village/town from Tunduru or Namtumbo districts	51
Outside of Tunduru or Namtumbo Districts	53
Considered as the most conflictive wildlife	
Elephant	365
Bushpig	75
Baboon	10
Hyena	8
Lion	7
Vervet monkey	7
Eland	3
Leopard	2
Impala	1
Jackal	1
Warthog	1
