# Supplementary material for: Let Us Give Voice to Local Farmers: Preferences for Farm-Based Strategies to Enhance Human–Elephant Coexistence in Africa
Source: Animals (Basel). 2022 Jul 21;12(14):1867. doi: 10.3390/ani12141867 (PMC9311559; doi:10.3390/ani12141867)
Supplement: Supplementary file 1 [file animals-12-01867-s001.zip › animals-1814890-Table S2.pdf]

Table S2. Willingness to pay. Results of the random parameter logit model. (*** significance at 1% level; ** significance at 5% level, * significance at 10% level).

	Coefficient	Standard Error	Z	Prob. |z|>Z*	95%Condidence Interval
Chili-oil fences	11.062 ***	1.2306	8.99	<0.001	(8.650, 13.4735)
Bee-hive fences	6.460 ***	1.2557	5.14	<0.001	(3.9991, 8.9212)
Technical support	6.001 ***	0.7716	7.78	<0.001	(4.4885, 7.5131)
Cooperation in big groups	3.990 ***	0.5976	6.68	<0.001	(2.8184, 5.1607)
Crop selection	2.630 **	1.1090	2.37	0.018	(0.4560, 4.8032)
Cooperation in small groups	-0.217	0.5476	-0.40	0.692	(-1.2901, 0.8565)
Noisemakers	-0.687	1.2157	-0.57	0.572	(-3.0700, 1.6953)
Crop translocation	-3.529 ***	1.0826	-3.26	0.001	(-5.6507, -1.4068)
Not doing anything	-22.670 ***	2.4599	-9.22	<0.001	(-27.491, -17.849)
